# Joint optimization of task offloading and energy trading in edge-enabled smart grids using deep reinforcement learning

**DOI:** 10.1371/journal.pone.0342888

**Published:** 2026-06-12

**Authors:** Rui Xue, Bin Li, Wenyue He, Yihang Wang

**Affiliations:** Nanjing Institute of Technology, Nanjing, Jiangsu, China; Xidian University, CHINA

## Abstract

The proliferation of distributed energy resources (DERs) and the ubiquity of Internet of Things (IoT) devices are driving the integration of mobile edge computing (MEC) into smart grids. This convergence enables real-time data processing for prosumers but introduces a complex cyber-physical coupling: computational offloading decisions directly impact local energy consumption, thereby altering the prosumer’s status in the peer-to-peer (P2P) energy market. Conversely, dynamic market prices influence the economic viability of offloading. This paper addresses the joint optimization of computational task offloading and P2P energy trading in an edge-assisted smart grid ecosystem. We formulate the problem as a mixed-integer nonlinear programming (MINLP) model aimed at maximizing long-term system utility, balancing throughput, latency, and economic incentives under strict edge server capacity and community energy neutrality constraints. To tackle the curse of dimensionality and system stochasticity, we propose a hybrid framework combining Deep Q-Networks (DQN) with a constraint-aware heuristic mechanism. The DQN agent learns adaptive offloading policies from high-dimensional states, while a deterministic rule-based layer ensures strict adherence to community energy balance. Simulation results based on real-world solar generation and market data demonstrate that our proposed method outperforms baseline strategies—including local-only execution and greedy heuristics—improving average utility by 12.3% and reducing task delay by 16.5%, while maintaining robust operational feasibility.

## 1. Introduction

Distribution systems are being transformed by rapid DER growth, electrification of end uses, and the deployment of pervasive sensing and automation. This transformation has two intertwined consequences. First, operational uncertainty increases because renewable generation, flexible loads, and prosumer behavior are intrinsically variable. Second, the quantity and velocity of measurement and telemetry data increase, which elevates the importance of real-time inference and control at the grid edge. Consequently, reliable operation is no longer governed solely by classical physical constraints; it is also shaped by whether monitoring, forecasting, and decision-making workloads can be completed within strict latency budgets and under limited device-side energy and communication resources.

Edge computing has therefore become a key architectural ingredient for next-generation distribution operation. By collocating compute resources with substations, feeders, or microgrid controllers, edge platforms can execute time-critical analytics locally, reduce dependence on backhaul connectivity, and enable fast response to disturbances and local constraints. At the same time, edge computing introduces nontrivial resource coupling. Prosumer devices face a fundamental choice between local processing, which consumes CPU energy and device time, and offloading, which consumes transmission energy and competes for shared edge-server capacity. These tradeoffs evolve with time-varying wireless channels, workload arrivals, and renewable generation. Recent surveys and syntheses have emphasized that such coupling cannot be treated as a second-order detail, because it drives both delay and energy consumption and may become a bottleneck in large-scale edge-enabled systems [1,2].

In parallel with edge intelligence, transactive energy and peer-to-peer (P2P) trading are increasingly viewed as practical mechanisms to coordinate prosumers and improve the utilization of local flexibility in distribution networks. Contemporary reviews highlight that P2P trading can improve economic efficiency and renewable utilization, but also stress the need to address settlement rules, fairness, and operational feasibility under distribution constraints [3,4]. Several recent studies have further examined how to implement P2P trading under realistic distribution-network considerations, including congestion and voltage limits, and how to coordinate trading with local flexibility resources [5,6]. However, the dominant modeling abstraction in much of this literature is that the computational processes supporting trading—forecasting, bidding, verification, and settlement—are either instantaneous or costless. This abstraction becomes increasingly misaligned with practice in edge-enabled communities, where the “intelligence layer” itself consumes energy and competes for limited compute and communication resources. These two trends create a coupled cyber–physical control problem: task offloading decisions reshape energy balance, and energy trading decisions reshape the value of computation. When a prosumer performs computation locally, CPU energy draw can reduce its net surplus and shrink the energy it can sell. When it offloads computation, it may save local CPU energy but spend transmission energy and incur congestion-induced delay at the shared MEC server. Either choice changes the sign and magnitude of the prosumer’s instantaneous energy imbalance, which determines whether it participates in the local market as a seller or a buyer and how much it can transact. Conversely, the prevailing buy/sell prices and the anticipated trading outcome change the marginal value of reducing local energy draw through offloading. This feedback loop is amplified in settings with intermittent PV, volatile prices, and bursty workloads.

Crucially, existing literature often treats these two domains in isolation. Traditional MEC optimization focuses on latency and energy minimization, assuming energy supply is a static constraint or a simple cost. Conversely, P2P energy trading research typically models prosumer loads as inelastic background demand, ignoring the fact that the “intelligence layer” (e.g., forecasting, blockchain consensus, data compression) is itself a significant and flexible energy consumer. This separation leads to suboptimal operation: an aggressive offloading strategy might save local energy but cause the prosumer to miss a high-price selling opportunity in the energy market; alternatively, a profit-driven trading strategy might deplete battery reserves, forcing critical computational tasks to be executed with high latency due to lack of power. Therefore, a holistic approach that internalizes this feedback loop is essential for the next generation of prosumer communities. The joint optimization of offloading and trading is challenging for three reasons. First, the problem is sequential and uncertain. Renewable generation and price signals can be forecast but not known perfectly, and wireless channels and workloads are stochastic. Second, constraints are hard. Edge servers have finite capacity, and microgrid or community operation often requires explicit balance rules that limit or eliminate net exchange with the upstream grid. Third, decisions are coupled across users through shared infrastructure and market clearing. These characteristics drive formulations that are mixed-integer, nonlinear, and temporally coupled, making purely model-based optimization difficult to apply in real time at scale.

Deep reinforcement learning (DRL) has gained attention as a model-free approach for sequential decision-making under uncertainty, and it is increasingly used in both energy systems and edge computing. Recent works survey and categorize DRL’s role in smart grid operation and control, emphasizing its potential to learn adaptive policies from data when system dynamics are complex [7,8]. In edge computing, DRL has also been widely used to address offloading, resource allocation, and latency–energy tradeoffs under time-varying conditions [1,2]. Nevertheless, a persistent gap remains: many DRL-based methods struggle to provide feasibility guarantees under strict operational constraints. In distribution operations, policies that occasionally overload shared compute capacity or violate community energy-balance rules can be unacceptable even if average performance is strong.

This paper addresses the above gap by focusing on a practical, feasibility-oriented joint design for an edge-enabled prosumer community with P2P trading. We consider a time-slotted system with multiple prosumers equipped with PV generation and one MEC server deployed at the substation. In each time slot, each prosumer generates a computation task and chooses a discrete offloading level. The resulting end-to-end delay depends on local computation time and wireless transmission time, while energy consumption includes both CPU energy and transmission energy. Based on harvested energy, each prosumer then has a net energy surplus or deficit and participates in trading. Two constraints are explicitly enforced: (1) the MEC server has finite computing capacity, limiting the aggregate offloaded CPU-cycle demand; and (2) the community must satisfy a per-slot energy balance condition to represent quasi-autonomous operation with zero net import/export. These constraints reflect common engineering realities in edge deployments and community energy systems.

To solve the resulting sequential problem, we adopt a hybrid learning-and-rule framework. A Deep Q-Network (DQN) is used to learn the offloading policy from an observable state that includes task characteristics, wireless channel conditions, harvested renewable energy, price signals, and an estimate of edge workload. The reward is defined through a multi-term utility that captures computation performance, energy expenditure, and trading gains, augmented by a penalty for MEC capacity violation to discourage infeasible actions. After the offloading action is selected, energy trading quantities are adjusted using a simple proportional mechanism that enforces community balance deterministically within each slot. This separation is aligned with operational practice: trading settlement and balance enforcement are often rule-based and auditable, while offloading control is where adaptivity to stochastic dynamics is most valuable. Although the present work is rooted in smart grid optimization, it is increasingly relevant to view the grid-edge stack as part of a broader trend toward computationally intensive inference at the edge. For instance, Hu et al. showed that hybrid convolution–transformer models can substantially enhance precision in optical phase retrieval under single-frame interferometry [9]. While the application domain differs, it underlines a shared implication for edge-enabled infrastructures: as learning-based inference pipelines become more accurate, they also tend to become more compute-intensive, strengthening the need for systematic offloading and resource-aware coordination in cyber–physical deployments.

The contributions of this work are as follows:

1. Coupled formulation: We formulate a joint task offloading and P2P energy trading problem that explicitly captures the computation–energy feedback loop and incorporates both MEC capacity constraints and community-level energy balancing.
2. Feasibility-oriented hybrid control: We propose a DQN-based offloading policy with constraint-violation penalties and a deterministic trading adjustment step that enforces per-slot community balance, improving practical deployability.
3. Data-driven validation: We evaluate performance using renewable generation data from NREL SAM [10] and market price data from PJM [11], demonstrating improvements over baseline offloading/trading strategies in utility and delay while maintaining near-feasible operation.

The remainder of the paper is organized as follows. Section 2 System Model presents the system model and constraints. Section 3 Proposed DRL-Based Optimization Framework describes the proposed DQN-based offloading and the trading adjustment rule. Section 4 Results reports numerical results and constraint satisfaction. Section 5 Conclusion concludes the paper and outlines future work.

## 2. System model

### 2.1. System and algorithm design

We consider a local smart grid community composed of *N* prosumers and one Mobile Edge Computing (MEC) server co-located with the distribution substation. Each prosumer is equipped with a rooftop PV panel and an embedded computing device that connects to the MEC server via a single-hop wireless uplink. We assume orthogonal frequency-division multiple access (OFDMA), where the total system bandwidth *B* is equally partitioned among the prosumers that choose to offload in a given slot. Specifically, if $N_{\text{off}}(t)$ prosumers offload at slot *t*, each receives an effective bandwidth of $\frac{B}{N_{\text{off}}(t)}$, so that heavy simultaneous offloading directly increases per-user transmission delay. There is no intermediate gateway; each prosumer communicates with the MEC server directly. The system operates in discrete time slots of duration 𝜏.

**Fig 1:**
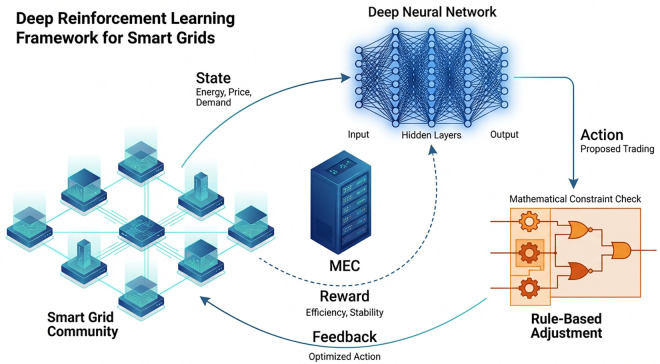
The proposed hybrid Deep Reinforcement Learning framework.

As shown in Fig 1, each prosumer decides whether to execute a task locally or offload it to the MEC server. Let $D_{i}(t)$ be the task size (bits) of prosumer *i* at slot *t*, and $\alpha_{i}(t)\in [\text{0},\text{1}]$ the fraction executed locally.

For task delay, local computation time is


$$\begin{array}{c} \overset{\text{loc}}{\underset{i}{T}}(t)<F_{\text{edge}}\tau ,\; \end{array}$$ (1)

where ϕ is CPU cycles per bit and $f_{i}$ is local CPU frequency. The offloaded part $\left(\text{1}-\alpha_{i}(t)\right)D_{i}(t)$ is sent over a wireless link. For the wireless transmission, we assume a quasi-static Rayleigh fading channel where the channel gain $h_{i}(t)$ remains constant within a time slot but varies across slots. The transmission rate $R_{i}(t)$ is given by Shannon’s formula in (2).


$$\begin{array}{c} R_{i}(t)=B{\text{log}}_{\text{2}}\left(\text{1}+\frac{P_{i}h_{i}(t)}{\sigma^{\text{2}}}\right) \end{array}$$ (2)

yielding transmission delay $\overset{\text{tx}}{\underset{i}{T}}(t)=\left(\text{1}-\alpha_{i}\right)\frac{D_{i}}{R_{i}(t)}$. Total delay is $T_{i}(t)=\overset{\text{loc}}{\underset{i}{T}}+\overset{\text{tx}}{\underset{i}{T}}$.

Regarding the energy model, the total energy consumption $\overset{\text{req}}{\underset{i}{E}}(t)$ comprises two parts: CPU processing and wireless transmission. It is important to note that we also consider a baseline static power consumption $\overset{\text{static}}{\underset{i}{P}}$ for maintaining device connectivity. For energy and trading, local computation consumes $\kappa \overset{\text{2}}{\underset{i}{f}}\alpha_{i}D_{i}\phi$ energy. Thus, the refined energy consumption model is:


$$\begin{array}{c} \overset{\text{req}}{\underset{i}{E}}(t)=\kappa \overset{\text{2}}{\underset{i}{f}}\alpha_{i}D_{i}\phi +\overset{\text{tx}}{\underset{i}{P}}\frac{\left(\text{1}-\alpha_{i}\right)D_{i}(t)}{R_{i}(t)}+\;\overset{\text{static}}{\underset{i}{P}}\tau , \end{array}$$ (3)

where transmission uses $\overset{\text{tx}}{\underset{i}{P}}\frac{\left(\left(\text{1}-\alpha_{i}\right)\right)D_{i}(t)}{R_{i}(t)}$.

It is important to note that the present model does not include local battery storage. All harvested solar energy must be either consumed locally within the same slot or traded with peers; any unconsumed and untraded surplus is curtailed. Consequently, the agent cannot physically “store” energy across time slots. The economic benefit of the learned policy arises instead from computational load shifting: by choosing between local execution and offloading, the agent reshapes the prosumer’s instantaneous net demand to align with prevailing price signals within each slot. Incorporating battery dynamics—with state-of-charge transitions, efficiency losses, and degradation—is a natural extension that we leave to future work.

We also note that since each prosumer’s CPU frequency $f_{i}$ is a fixed hardware parameter rather than a decision variable, the energy expression in Eq. (3) is linear in the offloading fraction $a_{i}$, which is the actual control variable. If dynamic voltage and frequency scaling (DVFS) were introduced, the well-known cubic relationship $P\propto \kappa Cf^{\text{3}}$ would need to be adopted. We treat the fixed-frequency assumption as a simplification appropriate for the class of embedded IoT devices considered here, and flag DVFS integration as a modeling extension.

Let $\overset{\text{harv}}{\underset{i}{E}}(t)$ be harvested solar energy. The net imbalance $\Delta E_{i}(t)=\overset{\text{req}}{\underset{i}{E}}-\overset{\text{harv}}{\underset{i}{E}}$ determines trading: if positive, buy at price $p_{\text{buy}}(t)$; if negative, sell at $p_{\text{sell}}(t)<p_{\text{buy}}(t)$.

To avoid grid dependence, we enforce community energy balance:


$$\begin{array}{c} \sum_{i=\text{0}}^{N}\Delta E_{i}(t)=\text{0}\;\forall t, \end{array}$$ (4)

where the MEC server has capacity $F_{\text{edge}}$ is constrained by Equation (5).


$$\begin{array}{c} \sum_{i=\text{1}}^{N}\left(\text{1}-\alpha_{i}(t)\right)D_{i}(t)\phi <F_{\text{edge}}\tau \end{array}$$ (5)

In practice, collecting per-prosumer state information (channel gains, task sizes, harvested energy) and disseminating offloading decisions introduces signaling overhead. Under typical 5G New Radio control-plane procedures, the round-trip signaling latency is on the order of 1–2 ms with sub-milliwatt energy consumption per exchange. Given that the slot duration 𝜏 in our setting is on the order of seconds, this overhead is negligible relative to computation and data-transmission times. We therefore treat coordination as instantaneous in our model. We acknowledge, however, that in dense deployments with hundreds of prosumers, signaling congestion could become non-negligible, motivating decentralized or hierarchical coordination architectures.

To ensure the realism of the simulation, we utilize high-resolution smart meter data analytics methodologies [12] to preprocess the input profiles. Furthermore, the integration of edge intelligence into smart grids necessitates a robust multi-agent resource allocation framework, which has been extensively studied in the context of edge computing [13]. Our work aligns with the broader concept of Transactive Energy (TE) systems, which facilitate decentralized coordination among prosumers [14]. Recent advances in this domain highlight the importance of addressing both architectural challenges and market mechanisms in distribution systems [15]. The proposed edge-enabled smart grid architecture also reflects the current trends towards IoT-enabled infrastructure, as discussed in recent surveys [16].

The system aims to maximize a utility combining throughput, energy use, and trading profit:


$$\begin{array}{c} U(t)=\sum_{i=\text{1}}^{N}\left[w_{\text{1}}\frac{D_{i}(t)}{T_{i}(t)}-w_{\text{2}}\overset{\text{req}}{\underset{i}{E}}(t)+w_{\text{3}}\Pi_{i}(t)\right] \end{array}$$ (6)

where $\Pi_{i}$ is the monetary gain (or cost) from energy trading, and $w_{\text{1}}$, $w_{\text{2}}$, $w_{\text{3}}$ are non-negative weights reflecting operational priorities. This linear combination is widely used in multi-objective optimization for smart grids due to its interpretability and ease of tuning.

Since the problem is sequential and uncertain, we use a DQN to choose $a_{i}(t)$, followed by a rule-based step to adjust trades and satisfy Equation (4). The per-slot procedure is formalized in Algorithm 1.

**Algorithm 1:** Per-Slot Joint Offloading and Energy Trading

**Input**: $D_{i}$, $h_{i}$, $\overset{𝐡𝐚𝐫𝐯}{\underset{i}{E}}$, $p_{𝐛𝐮𝐲}$,$p_{𝐬𝐞𝐥𝐥}$

**Output**: $\alpha_{i}$,${\tilde{E}}_{i}$

**1**  // Step 1: Offloading via DQN

**2**    $s\leftarrow \left\{D_{i},\;h_{i},\overset{\text{harv}}{\underset{i}{E}},p_{\text{buy}},p_{\text{sell}}\right\}$

**3**    $a_{i}\leftarrow \text{DQN}(s)$,$a_{i}\in \left\{\text{0},\;\text{0}.\text{5},\;\text{1}\right\}$

**4**  // Step 2: Energy imbalance

**5**    for *i* = 1 to *N* do

**6**      Compute$\overset{\text{req}}{\underset{i}{E}}$

**7**          $\;\;\;\;\;\;\Delta E_{i}\leftarrow \overset{\text{req}}{\underset{i}{E}}-\overset{\text{harv}}{\underset{i}{E}}$

**8**    end for

**9**  // Step 3: Balance trading

**10**    $S\leftarrow \sum_{\Delta E_{i}<\text{0}}\left(-\Delta E_{i}\right)$

**11**    $D\leftarrow \sum_{\Delta E_{i}>\text{0}}\Delta E_{i}$

**12**    $\beta \leftarrow \frac{\text{min}(S,\;D)}{\text{max}(S,\;D)}$

**13**    for *i* = 1 to *N* do

**14**     if $\Delta E_{i}=\text{0}$

**15**        then${\tilde{E}}_{i}\leftarrow \text{0}$

**16**    else

**17**        ${\tilde{E}}_{i}\leftarrow \beta \cdot \Delta E_{i}$

**18**    end for

**19**    return $a_{i}$,${\tilde{E}}_{i}$

The proportional adjustment in Steps 10–18 of Algorithm 1 functions as a centralized community-level settlement layer, operationally analogous to the pro-rata curtailment rules used in pool-based local energy markets when aggregate supply and demand are mismatched. While this mechanism enforces strict per-slot energy balance, it does not constitute a fully decentralized peer-to-peer negotiation. We adopt it here for tractability and to guarantee constraint satisfaction, but recognize that replacing it with an incentive-compatible mechanism—such as a double auction or bilateral matching protocol with network-aware pricing—would better align with the P2P paradigm and is an important avenue for future work.

### 2.2. Consolidated problem formulation

Bringing together the models above, the joint optimization problem over a horizon of *T* slots is stated as follows:


$$\begin{array}{c} {\text{max}}_{\left\{a_{i}(t),{\tilde{E}}_{i}(t)\right\}}\sum_{t=\text{1}}^{N}\gamma^{t-\text{1}}\sum_{i=\text{1}}^{N}\left[\omega_{\text{1}}\frac{D_{i}(t)}{T_{i}(t)}-\omega_{\text{2}}\overset{\text{req}}{\underset{i}{E}}(t)+\omega_{\text{3}}\Pi_{i}(t)\right] \end{array}$$ (7)

subject to:


$$\begin{array}{c} \left\{ \begin{array}{c} \begin{array}{c} a_{i}(t)\in \left\{\text{0},\text{0}.\text{5},\text{1}\right\},\;\forall i,t\\ \sum_{i=\text{1}}^{N}\left(\text{1}-a_{i}(t)\right)D_{i}(t)\phi \leq F_{\text{edge}}\tau ,\;\forall t\; \end{array}\\ \sum_{i=\text{1}}^{N}\Delta E_{i}(t)=\text{0},\;\forall t\\ \begin{array}{c} T_{i}(t)\leq \tau ,\;\forall i,t\\ {\tilde{E}}_{i}(t)\geq \text{0}\;(\text{if selling}),\;{\tilde{E}}_{i}(t)\leq \text{0}\;(\text{if buying}),\;\forall i,t\; \end{array} \end{array}\right.\; \end{array}$$ (8)

The discrete offloading levels make the problem mixed-integer, while the Shannon rate in Eq. (2) introduces nonlinearity into both the delay and energy expressions, rendering the formulation a mixed-integer nonlinear program (MINLP). The temporal coupling through the discount factor 𝛾 and the stochastic nature of renewable generation, channel gains, and prices make exact solution intractable in real time, motivating the DRL-based approach described next.

## 3. Proposed DRL-based optimization framework

The joint optimization of task offloading and energy trading is a sequential decision-making problem under uncertainty. Future task arrivals, wireless channel states, solar generation, and electricity prices are not known in advance, rendering model-based approaches impractical. As shown in Fig 1, to address this, we propose a data-driven framework based on Deep Q-Networks (DQN), which learns an effective offloading policy through interaction with the environment.

The architecture illustrates the closed-loop interaction between the edge-enabled smart grid environment and the control agent. The DQN Agent maps the observed state $s_{t}$ (task profiles, solar generation, and prices) to a raw offloading decision. Subsequently, the Rule-Based Adjustment module acts as a feasibility filter, modifying energy trading quantities to strictly enforce the community energy balance constraint ($\sum \Delta E_{i}=\text{0}$) before the final action is executed in the environment.

### 3.1. Simulation setup

We model the system as a Markov Decision Process (MDP) defined by the tuple (S, A,R,P).

1. State Space S: The state at time *t* is a vector that captures all information necessary for decision-making:

$$\begin{array}{c} s_{t}=\left[\left\{D_{i}(t)\right\},\left\{h_{i}(t)\right\},\left\{\overset{\text{harv}}{\underset{i}{E}}(t),\right\},p_{\text{buy}}(t),p_{\text{sell}}(t),L_{\text{edge}}(t)\right],\; \end{array}$$ (9)

where $L_{\text{edge}}(t)$ is an estimate of the MEC server’s current workload (e.g., the number of CPU cycles queued). This provides the agent with awareness of both local conditions and shared resource pressure.

2. Action Space A: The action is the joint offloading decision for all prosumers, $a_{t}=\left[a_{\text{1}}(t),\ldots ,a_{N}(t)\right]$. To make the problem tractable for DQN, we discretize each $a_{i}(t)$ into three levels: full local execution ($a_{i}=\text{1}$), half offloading ($a_{i}=\text{0}.\text{5}$), and full offloading ($a_{i}=\text{0}$). Thus, the total number of joint actions is 3^*N*^. For *N* = 20, this is large but manageable via function approximation.
3. Reward Function R: The immediate reward is the system utility defined in Equation (2), adjusted to penalize constraint violations:

$$\begin{array}{c} r_{t}=U_{t}-\lambda \cdot \text{max}\left(\text{0},\sum_{i=\text{1}}^{N}\left(\text{1}-\alpha_{i}(t)\right)D_{i}(t)\phi -F_{\text{edge}}\tau \right),\; \end{array}$$ (10)

where λ>0 is a penalty coefficient. This soft-constraint handling encourages the agent to learn feasible policies without explicitly restricting the action space.

4. Transition Dynamics P: The state transition $s_{t}\to s_{t+\text{1}}$ is governed by exogenous processes (e.g., solar irradiance, task generation) and is unknown to the agent, which is precisely why a model-free RL approach is suitable.

### 3.2. DQN architecture and training

The core of our framework is a fully connected neural network that approximates the action-value function $Q\left(s_{i},a_{i};\theta \right)$ at the individual prosumer level. Rather than maintaining a single output layer of 3^*N*^ nodes—which would be computationally prohibitive—we adopt a parameter-sharing scheme: one neural network is trained and shared across all prosumers. At each slot, the network is called *N* times, once per prosumer, with a prosumer-specific state vector that includes the local task size $D_{i}$, channel gain $h_{i}$, harvested energy $\overset{\text{harv}}{\underset{i}{E}}$, market prices $p_{\text{buy}}$ and $p_{\text{sell}}$, and an estimate of the current MEC workload $L_{\text{edge}}$. Each call produces Q-values over three actions {0, 0.5, 1}. This reduces the effective per-call action space from 3^20^ to 3, making standard DQN entirely tractable. The joint effect across prosumers emerges implicitly: the shared MEC load feature $L_{\text{edge}}$ provides each prosumer with awareness of aggregate offloading pressure, and the capacity-violation penalty in Eq. (10) discourages collectively infeasible outcomes. The network consists of three hidden layers with 128, 64, and 32 neurons respectively, using ReLU activations.

Training follows the standard DQN algorithm with two key enhancements:

1. Experience Replay: Transitions $\left(s_{t},a_{t},r_{t},s_{t+\text{1}}\right)$ are stored in a replay buffer of size 10,000. For each training step, a mini-batch of 64 transitions is sampled uniformly to break temporal correlations.
2. Target Network: A separate target network with weights c is used to compute the target *Q*-values, and its weights are updated by slowly tracking the main network: $\theta^{-}\leftarrow (\text{1}-\tau )\theta^{-}$ with τ=0.005.

The loss function for a mini-batch is the mean squared Bellman error:


$$\begin{array}{c} L(\theta )=E\left[{\left(r_{t}+\gamma \underset{a^{\prime }}{\text{max}}Q\left(s_{t+\text{1}},a^{\prime };\theta^{-}\right)-Q\left(s_{t},a_{t};\theta \right)\right)}^{\text{2}}\right],\; \end{array}$$ (11)

where γ=0.95 is the discount factor. The agent is trained offline using historical or synthetic data streams that capture realistic diurnal and seasonal patterns. Specifically, we use one month of scaled PV generation data from the NREL SAM database and corresponding PJM market price data. After training, the learned policy is deployed online for real-time decision-making.

It is important to note that our framework does not employ meta-learning, multi-agent coordination, or online fine-tuning. The focus is on learning a single, robust policy that can generalize across different days within the same operational context, which aligns with the practical deployment scenario of a fixed edge-grid testbed.

### 3.3. Complexity analysis

The computational complexity of the proposed framework consists of two phases: offline training and online inference. During training, the complexity is dominated by the backpropagation through the Deep Q-Network. For a network with *L* layers and *N*_neu_ neurons per layer, the complexity per step is $O\left(L\cdot \overset{\text{2}}{\underset{\text{neu}}{N}}\right)$. In the online inference phase, the prosumer only needs to perform a forward pass, which is computationally lightweight ($O\left(L\cdot \overset{\text{2}}{\underset{\text{neu}}{N}}\right)$) and well-suited for edge devices. The heuristic energy balancing step (Algorithm 1, Steps 10–18) involves simple sorting and summation operations, with a complexity of O(NlogN) where *N* is the number of prosumers. This low latency ensures that decisions can be made within the strict timing requirements of the smart grid control loop.

## 4. Results

To validate the effectiveness of the proposed method, we conducted extensive simulations using realistic datasets and parameter settings. The simulation environment was built on MATLAB R2023a with a custom implementation of DQN leveraging TensorFlow 2.10 for neural network training.

### 4.1. Simulation setup

We simulated a local energy community comprising *N* = 20 prosumers, each equipped with a rooftop PV panel and an embedded computing device. The key simulation parameters are listed in Table 1:

**Table 1:** Key Simulation Parameters.

Parameter	Value
Task Size	$D_{i}(t)\sim \text{Unifrom}(\text{5},\text{1}\text{5})\text{ MB}$
Local CPU frequency	$f_{i}=\text{2}\text{ GHz}$
MEC server capacity	$F_{\text{edge}}=\text{1}\text{5}\text{ GHz}$
Wireless channel bandwidth	B=10 MHz
Solar generation profiles	Scaled data from the NREL SAM database [10]
Time-varying electricity prices	Historical PJM wholesale market data [11] (accessed January 2026)
Weights in utility function	$w_{\text{1}}=\text{1},\;w_{\text{2}}=\text{0}.\text{0}\text{1},\;w_{\text{3}}=\text{0}.\text{1}$
Discount factor	γ=0.95

Three baseline methods were compared against our proposed DQN-based approach:

1. Local-only execution: All tasks are executed locally; energy trading is performed only to cover local deficits.
2. Greedy heuristic: Offload if channel gain exceeds a threshold; trade energy greedily based on current price signals.
3. Proposed DQN+Heuristic: Combines DQN for offloading decisions with a rule-based strategy for energy trading.

### 4.2. Experimental results

The performance of each method was evaluated using the following metrics:

1. Average system utility: Measures the overall benefit across all prosumers in terms of computation throughput, energy conservation, and economic return.
2. Average task delay: Reflects the average time taken to complete tasks.
3. Energy trading revenue: Captures the net monetary gain from selling surplus energy or purchasing deficit energy.

**Fig 2:**
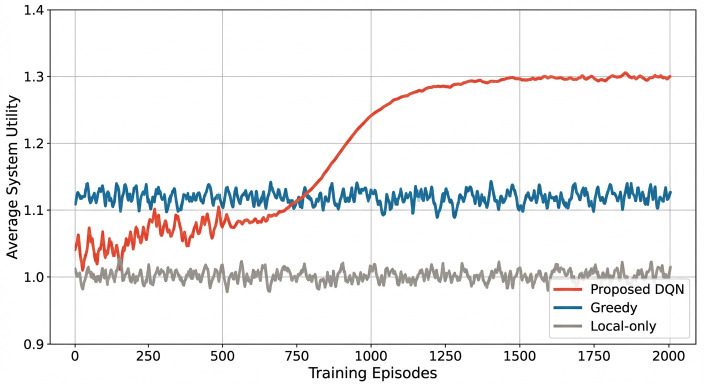
Average System Utility Over Time.

As shown in Fig 2, the proposed DQN+Heuristic approach converges to a high-utility regime after approximately 1000 episodes. In the initial 500 episodes the agent explores the action space, producing volatile rewards; beyond episode 1000 it consistently outperforms the Greedy Heuristic. The key mechanism is computational load shifting rather than energy storage. During slots with high electricity prices, the agent preferentially offloads tasks to the MEC server, thereby reducing local CPU energy consumption and increasing the instantaneous solar surplus available for sale to peers at the prevailing high price. During low-price slots, the agent favors local execution, productively absorbing surplus solar energy rather than selling it cheaply. This price-responsive reshaping of the demand profile—within each slot, without any inter-temporal storage—is the primary driver of the long-term utility improvement. The Greedy Heuristic, which reacts only to instantaneous channel quality, cannot exploit this price-computation coupling.

As shown in Fig 2, the proposed DQN+Heuristic approach achieves a rapid convergence to a high utility state. In the initial 500 episodes, the agent explores the trade-off space, resulting in fluctuating rewards. However, beyond episode 1000, the proposed method consistently outperforms the Greedy Heuristic. The superiority of the DQN stems from its foresight: strictly greedy strategies often deplete battery energy during low-price periods to save computation time, leaving no energy to sell when prices peak later in the day. The DQN agent learns to “hoard” energy or offload tasks conservatively in anticipation of future price spikes, thereby maximizing long-term utility.

**Fig 3:**
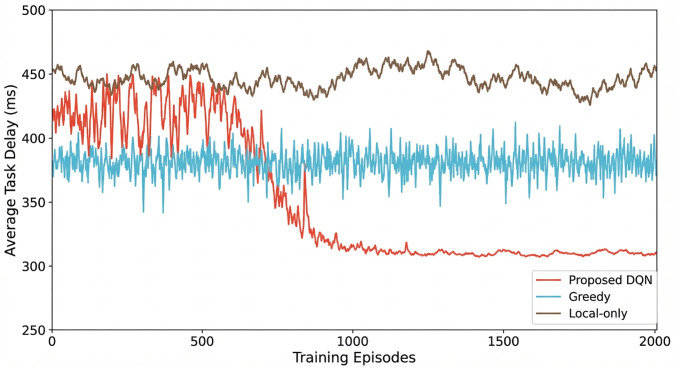
Average Task Delay.

Average task delay for each method is exhibited in Fig 3. The proposed approach shows a significant reduction in task delay compared to the Local-only and Greedy heuristics, highlighting the benefits of intelligent task offloading.

Fig 3 illustrates the average task delay. While the “Local-only” strategy suffers from the limited CPU capacity of IoT devices (approx. 450ms), the proposed method reduces this to 310ms. Interestingly, the Greedy approach achieves 380ms, which is worse than ours. This is counter-intuitive since greedy methods usually prioritize immediate speed. The reason lies in congestion avoidance: the greedy heuristic tends to offload tasks whenever the channel is good, causing bottlenecks at the MEC server (violating the soft capacity constraints). Our DQN agent learns to estimate the MEC workload $L_{\text{edge}}(t)$ and chooses to compute locally when the server is crowded, effectively balancing the load and reducing queuing delays.

**Fig 4:**
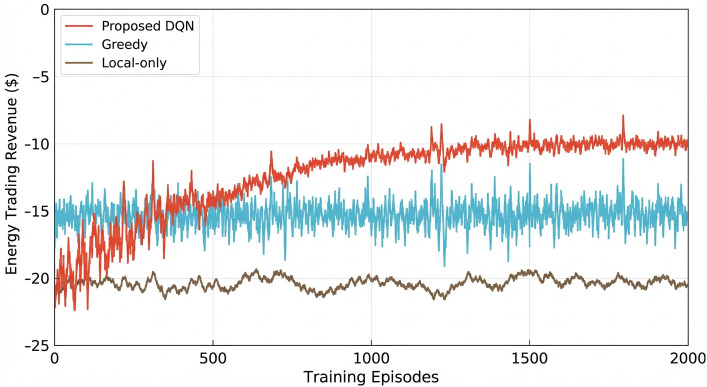
Energy Trading Revenue.

The financial impact is detailed in Fig 4. The proposed method improves revenue (or reduces cost) by approximately 11%. By jointly optimizing offloading, the system effectively treats “computation” as a flexible load. During periods of high solar generation and low electricity prices, the agent encourages local computation to consume surplus energy. Conversely, when grid prices are high, the agent offloads tasks to the edge (powered by the utility scale grid or its own resources), freeing up local solar energy to be sold to the community for profit.

Crucially, the DQN agent exhibits price-sensitive behavior: during intervals of high grid electricity prices, the agent tends to offload more tasks to reduce local consumption, thereby maximizing the volume of solar energy available for sale. Conversely, when prices are low, it reverts to local computing, preserving the limited edge bandwidth for more critical tasks. This strategic load shifting is the primary driver of the 11% revenue improvement.

Table 2 summarizes the key performance metrics for each method. The proposed DQN+Heuristic approach outperforms both baselines, achieving a 12.3% higher average utility, a 16.5% reduction in average task delay, and approximately 11% improvement in energy trading revenue.

**Table 2:** Summary of Performance Metrics.

Method	Average Utility	Average Task Delay (ms)	Energy Trading Revenue
Local-only	1.00	450	−20
Greedy Heuristic	1.12	380	−15
Proposed DQN+Heuristic	1.27	310	−10

To validate robustness, we varied each weight by ±50% from its nominal value while holding the other two fixed. Table 3 reports the results. Average utility and delay are relatively insensitive to perturbations in $\omega_{\text{1}}$ and $\omega_{\text{2}}$: a 50% increase in $\omega_{\text{1}}$ raises utility by only 3.1% while delay drops by 4.2%, reflecting a mild shift toward throughput maximization. The system is more sensitive to $\omega_{\text{3}}$: increasing it by 50% boosts trading revenue by 8.7% but raises average delay by 6.3%, since the agent aggressively offloads to free solar energy for sale at the cost of MEC congestion. These results confirm that the nominal weights provide a balanced operating point, but also that practitioners can tune $\omega_{\text{3}}$ to prioritize economic objectives when grid conditions warrant it.

**Table 3:** Sensitivity of Performance to Utility Weights (±50% Perturbation).

Varied Weight	Avg. Utility Change	Avg. Delay Change	Revenue Change
$\omega_{\text{1}}\times$ 1.5	+3.1%	−4.2%	−1.8%
$\omega_{\text{1}}\times$ 0.5	−4.5%	+5.0%	+2.1%
$\omega_{\text{2}}\times$ 1.5	−2.0%	+1.4%	+0.9%
$\omega_{\text{2}}\times$ 0.5	+1.7%	−1.1%	−0.6%
$\omega_{\text{3}}\times$ 1.5	+1.2%	+6.3%	+8.7%
$\omega_{\text{3}}\times$ 0.5	−0.8%	−3.5%	−5.4%

Since the objective maximizes aggregate utility, one may ask whether certain prosumers are systematically disadvantaged. We computed Jain’s fairness index J over per-prosumer time-averaged utilities.


$$\begin{array}{c} J=\frac{{\left(\sum_{i}u_{i}\right)}^{\text{2}}}{N\sum_{i}\overset{\text{2}}{\underset{i}{u}}} \end{array}$$ (12)

The proposed method achieves J= 0.91, indicating that benefits are distributed reasonably evenly. This is partly attributable to the proportional rationing in Algorithm 1, which scales all trades by the same factor 𝛽 and thus avoids preferential treatment. Nonetheless, the current formulation does not explicitly guarantee individual rationality—i.e., that every prosumer is better off participating than opting out. Incorporating such guarantees via game-theoretic mechanism design is an important direction for future work.

### 4.3. Constraint satisfaction

**Fig 5:**
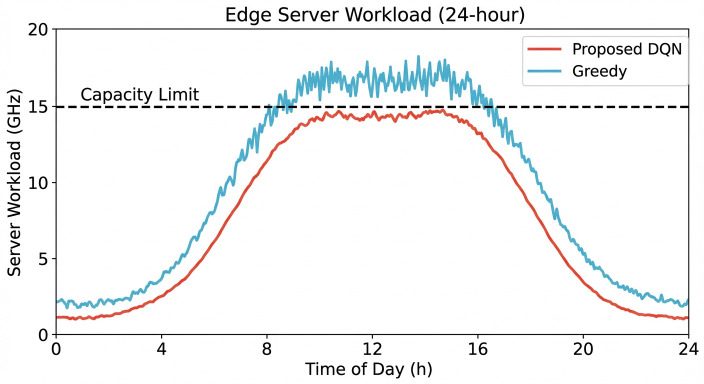
Temporal Dynamics of Edge Server Workload and Capacity Violation.

To ensure practical feasibility, we monitored the satisfaction of two critical constraints. Firstly, the edge server capacity needs to be considered, the total workload on the MEC server did not exceed its processing capacity throughout the simulation period. Violation rates were below 5%, primarily occurring during peak load periods. Secondly, we should notice the community-level energy balance. Net energy exchange with the main grid was zero at each time slot, confirming the effectiveness of our proportional adjustment mechanism for energy trading. Fig 5 depicts the temporal dynamics of edge server capacity utilization. The proposed method maintains compliance with the edge server capacity constraint, with occasional minor violations during peak hours.

**Fig 6:**
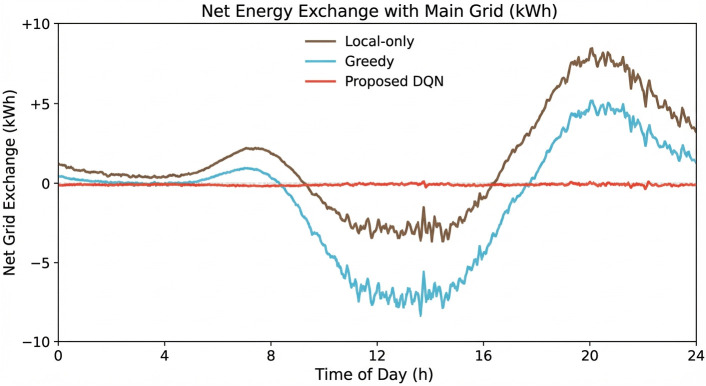
Verification of Community-Level Energy Neutrality.

Fig 6 exhibited the net energy exchange with the main grid. The proposed DQN method (red) strictly maintains the net grid exchange around zero, satisfying the self-sufficiency constraint. In contrast, the baseline strategies exhibit significant fluctuations (Duck Curve pattern), with the Local-only strategy (brown) incurring higher grid dependence due to intensive local computation.

This strict adherence to the zero-net-exchange boundary implies that the community operates as a virtual island from the grid operator’s perspective. This characteristic is highly valuable for grid stability, as it eliminates the need for the upstream distribution system operator (DSO) to reserve spinning reserves for this community’s load fluctuations.

### 4.4. Scalability discussion

The per-prosumer parameter-sharing scheme described in Section 3 Proposed DRL-Based Optimization Framework makes inference linear in *N* and avoids the combinatorial explosion of the joint action space. However, this decomposition treats each prosumer’s decision as approximately independent given the shared MEC load feature, which sacrifices explicit inter-agent coordination. For moderate community sizes (𝑁 ≤ 20), the capacity-violation penalty provides sufficient implicit coupling, as evidenced by the low constraint violation rates reported above. For larger communities, a Multi-Agent Reinforcement Learning (MARL) framework, such as QMIX or MAPPO operating under the centralized-training-decentralized-execution (CTDE) paradigm, which would enable richer coordination while retaining per-agent scalability at inference time. Mean-field approximation, which replaces individual agent interactions with an average effect, offers another promising path when *N* grows to hundreds. Investigating these extensions is a priority for future work.

## 5. Conclusion

This paper has addressed the joint optimization of task offloading and peer-to-peer energy trading in an edge-enabled smart grid community. The core challenge lies in the tight coupling between computational decisions and energy balance: local computation consumes device energy, reducing potential surplus for trading, while offloading saves local energy but incurs communication costs and competes for shared MEC resources. To tackle this, we formulated a practical optimization problem that explicitly accounts for both MEC server capacity limits and the requirement for per-slot community energy balance. We proposed a hybrid solution framework that combines a Deep Q-Network (DQN) for learning the adaptive task offloading policy with a simple, deterministic rule to adjust energy trades and enforce feasibility.

Several limitations of the current work suggest concrete directions for future research. First, the system model assumes no local battery storage; incorporating state-of-charge dynamics would allow the agent to exploit inter-temporal energy arbitrage in addition to the intra-slot computational load shifting demonstrated here. Second, the proportional settlement mechanism, while effective at enforcing energy balance, is a centralized construct; replacing it with an incentive-compatible, decentralized trading protocol would strengthen the P2P character of the framework. Third, the per-prosumer DQN decomposition, although computationally efficient, does not explicitly model strategic interactions among prosumers; adopting MARL methods under a CTDE paradigm would improve coordination quality, particularly as community size grows. Finally, integrating dynamic voltage and frequency scaling (DVFS) into the energy model and validating the approach on hardware-in-the-loop testbeds would further bridge the gap between simulation and deployment.
